# Laser assisted versus photodynamic decontamination of peri-implant mucositis: A split mouth RCT

**DOI:** 10.6026/973206300211103

**Published:** 2025-05-31

**Authors:** Randhir Kumar, Amit Goel, Ruchi Pandey, Arshad Jamal Sayed, Surya Dahiya, Jeethu John Jerry, Sukriti Mukherjee

**Affiliations:** 1 Department of Periodontics Patna Dental College and Hospital, Patna, Bihar, India; 2 Department of Dentistry, ShriSiddhivinayak Medical College, Sambhal, Moradabad, Utter Pradesh, India; 3 Department of Periodontology, ManavRachna Dental College, SDS, MRIIRS, Faridabad, Haryana, India; 4 Department of Periodontology and Implant Dentistry, College of Dentistry, Qassim University, Saudi Arabia; 5 Department of Conservative Dentistry and Endodontics, Maharishi Markandeshwar College of Dental Sciences and Research, Mullana (Ambala), Haryana, India; 6 Department of Periodontology, Malabar Dental College and Research Centre, Edappal, Malapuram, Kerala, India; 7 Department of Oral Medicine and Radiology, Kalinga Institute of Dental Sciences, KIIT Deemed to be University, Patia, Bhubaneswar-751024, Odisha, India

**Keywords:** Peri-implant mucositis, diode-laser assisted decontamination (DLAD), photodynamic therapy (PDT), platform-switch implants, laser therapy

## Abstract

The clinical efficacy of diode-laser assisted decontamination (DLAD) and Photodynamic Therapy (PDT) in managing peri-implant mucositis around platform-switch implants is of interest. A split-mouth randomized controlled trial (RCT) was conducted with 50 participants (100 sites), each receiving both DLAD and PDT on different sides of the mouth. Clinical parameters were measured before and after treatment at baseline, 1 month, 3 months and 6 months. Both DLAD and PDT are effective in treating peri-implant mucositis around platform-switch implants, with no significant difference in clinical outcomes.

## Background:

Peri-implant mucositis is a common inflammatory condition affecting the soft tissues surrounding dental implants [1]. It is primarily marked by symptoms such as bleeding on probing (BoP), increased probing depths and inflammation, but without associated bone loss [2]. If not addressed in its early stages, peri-implant mucositis can progress to peri-implantitis, a more severe form that involves both soft tissue inflammation and progressive bone loss, which can ultimately compromise implant stability and lead to implant failure [3]. As such, early diagnosis and appropriate management are essential to preserve implant health and ensure long-term success [4]. Conventional treatment options typically involve mechanical debridement and the use of antiseptic agents; however, adjunctive non-invasive therapies such as Diode-Laser Assisted Decontamination (DLAD) and Photodynamic Therapy (PDT) have recently emerged as promising alternatives. DLAD utilizes a diode laser to reduce microbial contamination on the implant surface and enhance tissue healing [5, 7]. PDT involves the activation of a photosensitizing agent with light to exert antimicrobial effects and stimulate tissue repair. Both approaches aim to reduce inflammation and bacterial load in a minimally invasive manner [7]. Despite individual studies reporting favorable outcomes with each technique, direct comparisons between DLAD and PDT in the context of peri-implant mucositis are limited, especially in cases involving platform-switch implants, which possess distinct anatomical features that may influence therapeutic response [7-8]. Therefore, it is of interest to assess the clinical efficacy of Diode-Laser Assisted Decontamination (DLAD) and Photodynamic Therapy (PDT) in managing peri-implant mucositis around platform-switch implants.

## Materials and Methods:

This study was done in Department of Periodontics and after approval from the institutional ethics committee and informed consent from the participants. This split-mouth randomized controlled trial (RCT) was designed to evaluate and compare the effectiveness of Diode-Laser Assisted Decontamination (DLAD) and Photodynamic Therapy (PDT) in managing peri-implant mucositis around platform-switch implants. The study involved 50 participants, each contributing two treatment sites for a total of 100 sites. Each patient received both treatments, one on each side of the mouth, ensuring intra-subject comparison. Participants included were between 25 and 70 years of age, all diagnosed with peri-implant mucositis around platform-switch implants and free from medical conditions known to impair healing. Individuals with peri-implantitis, systemic diseases such as uncontrolled diabetes, or contraindications to laser or photodynamic therapy were excluded. The clinical parameters evaluated included Bleeding on Probing (BoP), Probing Depth (PD) and Plaque Index (PI), assessed both before and after the intervention. BoP was scored from 0 to 2, with 0 indicating no bleeding and 2 indicating moderate bleeding; PD was recorded in millimetres; and PI ranged from 1 (minimal plaque) to 3 (heavy plaque accumulation). The DLAD protocol involved treating the peri-implant area with a diode laser set to specific wavelength and power settings to achieve decontamination. PDT involved applying a photosensitizing agent to the site, which was then activated using a light source at the appropriate wavelength to exert antimicrobial action. Clinical assessments were conducted immediately after the intervention and at subsequent follow-ups at 1 month, 3 months and 6 months to monitor changes in clinical outcomes. Statistical analysis included paired t-tests for within-group comparisons before and after treatment and independent t-tests for comparisons between the two treatment groups. A significance threshold of p < 0.05 was used to determine statistical relevance.

## Results:

The demographic profile of the participants in this study showed a slightly higher representation of females, comprising 54% of the total sample, while males accounted for 46%. In terms of implant location, 60% of the treated sites were in the upper jaw, with the remaining 40% located in the lower jaw. Participants ranged in age from 25 to 70 years, with the mean age being approximately 45 years (Table 1). The clinical parameters recorded before and after treatment demonstrated notable improvements in both treatment groups. For bleeding on probing (BoP), the mean score reduced from 1.6 ± 0.5 to 0.9 ± 0.7 in the DLAD group and from 1.5 ± 0.5 to 0.8 ± 0.6 in the PDT group. Probing depth (PD) also showed a decrease, with values declining from 4.6 ± 0.7 mm to 3.9 ± 0.5 mm following DLAD and from 4.6 ± 0.6 mm to 3.8 ± 0.6 mm after PDT. Similarly, the plaque index (PI) dropped from 2.0 ± 0.6 to 1.3 ± 0.5 in the DLAD group and from 2.1 ± 0.5 to 1.3 ± 0.6 in the PDT group (Table 2). In this study, treatment success was determined based on achieving a reduction of more than 1 point in Bleeding on Probing (BoP), more than 0.5 mm in Probing Depth (PD) and more than 0.5 in Plaque Index (PI). Both the Diode-Laser Assisted Decontamination (DLAD) and Photodynamic Therapy (PDT) groups achieved equal success rates, with 9 out of 50 sites in each group meeting the criteria. This corresponds to a success rate of 18% for both DLAD and PDT, indicating comparable clinical effectiveness between the two modalities in managing peri-implant mucositis under the defined parameters (Table 3). Both DLAD and PDT resulted in significant reductions in BoP, PD and PI within groups, but there was no significant difference between the two treatment modalities. The odds ratio of 1.0 further indicates equal efficacy in terms of treatment success (Table 4).

## Discussion:

Peri-implant mucositis is a reversible inflammatory condition that affects the soft tissues surrounding dental implants, characterized primarily by bleeding on probing (BoP), increased probing depths (PD) and plaque accumulation, without evidence of bone loss [9, 10]. Effective management of this condition is crucial to prevent its progression to peri-implantitis, which can ultimately compromise implant stability and success. Among various non-surgical treatment modalities, two minimally invasive approaches-Diode-Laser Assisted Decontamination (DLAD) and Photodynamic Therapy (PDT)-have shown promise in improving peri-implant tissue health [7, 8, 9, 10, 11- 12]. Diode-Laser Assisted Decontamination (DLAD) operates by emitting laser energy in a specific wavelength range (typically 810-980 nm) that effectively penetrates inflamed soft tissues. This energy induces bacterial cell wall destruction through photothermal effects, reducing the microbial load in the peri-implant sulcus. Additionally, the laser promotes bio stimulatory effects such as increased fibroblast proliferation, collagen formation and angiogenesis, all of which contribute to enhance soft tissue healing [12, 13]. Photodynamic Therapy (PDT), on the other hand, involves the application of a photosensitizing agent-commonly methylene blue or toluidine blue-followed by exposure to a low-power light source of a specific wavelength. Upon activation, the photosensitizer produces reactive oxygen species (ROS) that destroy bacterial cells without damaging host tissues. PDT has gained traction due to its selective targeting of pathogens, minimal risk of resistance and its additional benefits in modulating inflammation and supporting tissue regeneration [11, 14]. Both DLAD and PDT are advantageous for their minimally invasive nature, targeted action and potential to be used adjunctively with mechanical debridement [15]. In the context of platform-switch implants-which feature a narrower abutment-diameter interface and unique crestal bone preservation characteristics-choosing an effective treatment that respects the soft tissue and bone interface becomes particularly important [16]. This study builds on the premise that both DLAD and PDT can play a vital role in the conservative management of peri-implant mucositis and aims to compare their clinical efficacy in a controlled split-mouth design. The results of this study demonstrate that both Diode-Laser Assisted Decontamination (DLAD) and Photodynamic Therapy (PDT) are effective in managing peri-implant mucositis around platform-switch implants, with significant reductions observed in key clinical parameters, including Bleeding on Probing (BoP), Probing Depth (PD) and Plaque Index (PI). The findings suggest that both treatment modalities show similar efficacy in reducing signs of inflammation and improving peri-implant health, supporting the use of either method as an adjunctive therapy for peri-implant mucositis.

The reduction in BoP and PD observed in both groups aligns with previous studies that have highlighted the beneficial effects of laser-assisted treatments and PDT in reducing inflammation and pocket depth in peri-implant tissues. For instance, Lin *et al.* (2018) [17] demonstrated that laser therapy effectively reduces inflammation and BoP in peri-implant mucositis, a finding that is consistent with our study, where DLAD significantly reduced BoP by 0.7 units on average. Similarly, PDT has been shown in various studies to have an antimicrobial effect that reduces pocket depth and inflammation [18]. In the current study, PDT similarly reduced PD by an average of 0.8 mm, suggesting that both treatments help in improving peri-implant tissue health by addressing both bacterial load and inflammatory responses. The improvement in the Plaque Index (PI) seen in both groups is also noteworthy. While mechanical debridement remains a cornerstone in treating peri-implant mucositis, adjunctive therapies like DLAD and PDT can further enhance plaque control. Alasqah (2019) reported that PDT can significantly reduce bacterial load and improve plaque control around implants and our findings support this by demonstrating similar reductions in PI. Both treatment modalities showed an average reduction of 0.7-0.8 in PI, highlighting their role in improving oral hygiene around implants [19].

An important finding of this study is the lack of significant difference between DLAD and PDT in terms of treatment outcomes. While both therapies showed substantial improvements in clinical parameters, statistical analysis revealed no significant differences in their efficacy (p > 0.05). This is consistent with Sánchez-Martos *et al.* (2023) [5], who found that both laser therapy and PDT could achieve similar outcomes in reducing peri-implant mucositis symptoms. Both treatments aim to reduce microbial contamination and inflammation, which may explain why their clinical effects are comparable. Moreover, the success rate for both treatments was found to be 18%, which, while seemingly modest, reflects the challenging nature of treating peri-implant mucositis in a clinical setting. Barootchi *et al.* (2020) [20] suggested that successful treatment of peri-implant mucositis requires not only mechanical debridement but also a reduction in bacterial load, both of which are effectively addressed by DLAD and PDT. In the context of this study, the 18% success rate indicates that these therapies can play a role in managing peri-implant mucositis when used as adjuncts to traditional treatment approaches. The odds ratio calculated in this study was 1.0, indicating that both treatments had an equal likelihood of success. This further supports the notion that DLAD and PDT are comparable in their clinical outcomes.

## Clinical implications:

The findings of this study suggest that both DLAD and PDT are viable treatment options for managing peri-implant mucositis around platform-switch implants. Given the ease of application and minimal invasiveness of both therapies, they may be considered as adjunctive treatments alongside conventional approaches such as mechanical debridement and antimicrobial therapy. Clinicians can confidently use either modality, depending on patient needs and preferences, as both treatments offer comparable results in improving clinical parameters and reducing symptoms of peri-implant mucositis.

## Limitations and future research:

While this study provides valuable insights, it is important to acknowledge some limitations. The sample size was relatively small and the study duration was limited to 6 months. Longer follow-up periods and larger sample sizes are needed to fully assess the long-term efficacy of DLAD and PDT. Additionally, further research into the cost-effectiveness, patient satisfaction and potential side effects of these treatments would be beneficial for making informed clinical decisions.

## Conclusion:

Both diode-laser assisted decontamination (DLAD) and Photodynamic Therapy (PDT) are effective in managing peri-implant mucositis around platform-switch implants, with no significant difference in their clinical efficacy. These findings suggest that clinicians can consider either therapy as an adjunct to traditional treatment approaches for peri-implant mucositis, depending on individual patient needs.

## Figures and Tables

**Table 1:** The demographic data of the study participants is summarized as follows

**Demographic Parameter**	**Distribution**
Gender	Female: 54%, Male: 46%
Location	Upper Jaw: 60%, Lower Jaw: 40%
Age Range	25-70 years (mean age 45)

**Table 2:** Clinical parameters before and after treatment

**Parameter**	**DLAD Before**	**PDT Before**	**DLAD After**	**PDT After**
BOP (Bleeding on Probing)	1.6 ± 0.5	1.5 ± 0.5	0.9 ± 0.7	0.8 ± 0.6
PD (Probing Depth in mm)	4.6 ± 0.7	4.6 ± 0.6	3.9 ± 0.5	3.8 ± 0.6
PI (Plaque Index)	2.0 ± 0.6	2.1 ± 0.5	1.3 ± 0.5	1.3 ± 0.6
Note: Data shown as mean ± standard deviation

**Table 3:** Treatment success was defined as a reduction in BoP> 1, PD > 0.5 mm and PI > 0.5

**Treatment Group**	**Success Count**	**Success Rate (%)**
Diode-Laser Assisted Decontamination (DLAD)	Sep-50	18%
Photodynamic Therapy (PDT)	Sep-50	18%

**Table 4:** Odds ratio summary

**Treatment Group**	**Success Cases**	**Total Cases**	**Success Rate**	**Odds Ratio (vs. other group)**
DLAD	9	50	18%	1
PDT	9	50	18%	1
